# Dosage analysis of cancer predisposition genes by multiplex ligation-dependent probe amplification

**DOI:** 10.1038/sj.bjc.6602121

**Published:** 2004-09-10

**Authors:** D J Bunyan, D M Eccles, J Sillibourne, E Wilkins, N Simon Thomas, J Shea-Simonds, P J Duncan, C E Curtis, D O Robinson, J F Harvey, N C P Cross

**Affiliations:** 1 National Genetics Reference Laboratory (Wessex), Salisbury District Hospital, Salisbury SP2 8BJ, UK; 2 Wessex Regional Genetics Laboratory, Salisbury District Hospital, Salisbury SP2 8BJ, UK; 3 Wessex Clinical Genetics Service, Princess Anne Hospital, Southampton, UK

**Keywords:** *hMLH1*, *hMSH2*, *BRCA1*, *BRCA2*, *APC*, MLPA

## Abstract

Multiplex ligation-dependent probe amplification (MLPA) is a recently described method for detecting gross deletions or duplications of DNA sequences, aberrations which are commonly overlooked by standard diagnostic analysis. To determine the incidence of copy number variants in cancer predisposition genes from families in the Wessex region, we have analysed the *hMLH1* and *hMSH2* genes in patients with hereditary nonpolyposis colorectal cancer (HNPCC), *BRCA1* and *BRCA2* in families with hereditary breast/ovarian cancer (BRCA) and *APC* in patients with familial adenomatous polyposis coli (FAP). Hereditary nonpolyposis colorectal cancer (*n*=162) and FAP (*n*=74) probands were fully screened for small mutations, and cases for which no causative abnormality were found (HNPCC, *n*=122; FAP, *n*=24) were screened by MLPA. Complete or partial gene deletions were identified in seven cases for *hMSH2* (5.7% of mutation-negative HNPCC; 4.3% of all HNPCC), no cases for *hMLH1* and six cases for *APC* (25% of mutation negative FAP; 8% of all FAP). For *BRCA1* and *BRCA2*, a partial mutation screen was performed and 136 mutation-negative cases were selected for MLPA. Five deletions and one duplication were found for *BRCA1* (4.4% of mutation-negative BRCA cases) and one deletion for *BRCA2* (0.7% of mutation-negative BRCA cases). Cost analysis indicates it is marginally more cost effective to perform MLPA prior to point mutation screening, but the main advantage gained by prescreening is a greatly reduced reporting time for the patients who are positive. These data demonstrate that dosage analysis is an essential component of genetic screening for cancer predisposition genes.

Multiplex ligation-dependent probe amplification (MLPA) is a new, high-resolution method for detecting copy number variations in genomic sequences (Schouten *et al*, 2002). Available evidence suggests that MLPA is a robust assay, which offers several advantages over existing techniques (Taylor *et al*, 2003). Many diagnostic genetics laboratories are therefore adopting MLPA for analysis of genes such as *hMLH1*, *hMSH2*, *BRCA1*, *BRCA2* and *APC* in preference to other techniques, for example, those based on quantitative-fluorescent PCR (QF-PCR) or multiple amplifiable probe hybridisation (MAPH). Laboratories have to decide whether dosage analysis should be performed before or after point mutation analysis, a decision that depends in part on the frequency of copy number variants in the local population.

Copy number variants in cancer predisposition genes are found at a frequency of around 4–15% in most countries, although founder effects have been reported in some study populations (Petrij-Bosch *et al*, 1997; Wagner *et al*, 2003). Previous studies of hereditary nonpolyposis colorectal cancer (HNPCC) patients using QF-PCR or Southern blotting have detected *hMLH1* and *hMSH2* copy number variants in 5% of probands in Germany (Wang *et al*, 2003), 5% in Japan (Nakagawa *et al*, 2003), 15% in France (Charbonnier *et al*, 2002) and 27% in the USA (Wagner *et al*, 2003). Two analyses using MLPA have reported *hMLH1* and *hMSH2* copy number variants in 13% of families from Holland (Gille *et al*, 2002) and 6% from the North of England (Taylor *et al*, 2003). Multiplex ligation-dependent probe amplification studies of breast/ovarian cancer patients have given similar figures with 14% of Italian probands (Montagna *et al*, 2003) and 4% of Dutch probands (Hogervorst *et al*, 2003) having a dosage variant of the *BRCA1* gene. One published study of familial adenomatous polyposis coli (FAP) patients using QF-PCR detected large deletions of *APC* in 12% of classical polyposis patients (Sieber *et al*, 2002).

With such a significant detection rate of copy number variants, it is clearly important to decide whether MLPA should be employed before or after standard mutation analysis. We present here an analysis of the frequency of copy number variants in HNPCC, FAP and hereditary breast/ovarian cancer from the Wessex region of the UK (which has a population of approximately 3 million people) and compare the relative testing cost and waiting times for prospective *vs* retrospective MLPA screening.

## MATERIALS AND METHODS

### HNPCC cohort

During the 8-year period 1995–2003, a total of 162 probands referred for suspected HNPCC from the Wessex region were collected and screened for point mutations, microdeletions or microinsertions of the *hMLH1* and *hMSH2* genes using single-stranded conformation polymorphism (SSCP) heteroduplex analysis or, more recently, denaturing high-performance liquid chromotography (dHPLC) followed by direct sequencing of abnormal fragments. In all, 50 of these patients fulfilled the Amsterdam criteria for HNPCC, that is, three relatives with colorectal cancer (CRC), one of whom is a first-degree relative of the other two; CRC involving at least two generations; one or more CRC cases diagnosed before the age of 50 years. A causative mutation, that is, a premature stop codon, frameshift, splice recognition site change or amino-acid change that is known to compromise function, was found in a total of 40 patients (24.7%), of whom 24 fulfilled the stringent Amsterdam criteria (48% of Amsterdam-positive patients) and 16 were patients who fulfilled one or more of the less stringent Bethesda Criteria (14% of patients not fulfilling the Amsterdam Criteria). The 122 patients in whom no mutation had been found were subsequently analysed by MLPA.

### Hereditary breast/ovarian cancer cohort

Over the 6-year period 1997–2003, we performed a partial *BRCA1* and *BRCA2* screen on 679 probands. All patients were tested using the protein truncation test (PTT) to look for frameshift or nonsense mutations in exon 11 of both *BRCA1* and *BRCA2* and by SSCP or dHPLC followed by sequencing of aberrant fragments for *BRCA1* exons 2 and 20 plus *BRCA2* exon 10. This partial screen covers approximately 65 and 55% of the *BRCA1* and *BRCA2* coding sequences, respectively, and a causative mutation was found in 99 patients (15%). All pedigrees were reviewed and ranked in order of likelihood for an underlying *BRCA1* or *BRCA2* mutation using the recently developed Manchester scoring system (Evans *et al*, 2004). In brief, each breast or ovarian cancer scores according to the age at onset and cancer site (ovarian cancer scores higher than breast cancer), with a small additional score for prostate and pancreatic cancers in *BRCA2*. A score of 10 or more equates to at least a 15% chance of a mutation being detected, the higher the crude score, the higher the likelihood. In all, 259 samples scoring 10 or above had been partly or wholly analysed in our laboratory previously and 83 mutations had been detected (32%). In order to assess the usefulness of *BRCA1* and *BRCA2* MLPA, we analysed 136 cases selected from this group where DNA was available for further analysis and in which no mutation had been found in the previous screen.

### FAP cohort

During the 11-year period 1992–2003, a total of 74 FAP probands from the Wessex region were collected and screened for point mutations, microdeletions or microinsertions of the *APC* gene using SSCP or dHPLC followed by direct sequencing of abnormal fragments. A causative mutation was found in a total of 50 patients (68%). The 24 patients in whom no mutation had been found were subsequently analysed by MLPA.

### Multiplex ligation-dependent probe amplification

**Figure 1:**
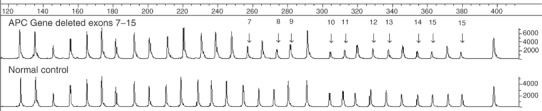
An example of the genotyper output from a patient with a deletion of exons 7–15 of the APC gene when compared to a normal individual.

Multiplex ligation-dependent probe amplification analysis was performed using kits from MRC-Holland (Amsterdam, The Netherlands). The kits include probes for each exon of the gene in question (except BRCA2 exons 5, 6, 23 and 26), probes for a number of control regions across the genome, plus further controls to check for adequate quality of test DNA and efficient ligation. The only alteration to the manufacturer's protocol was that 1 *μ*l of DNA was used per MLPA reaction (typically 400–600 ng) rather than the suggested 100 ng of DNA. Multiplex ligation-dependent probe amplification PCR products were separated on an ABI3100 genetic analyser and interpreted using Genotyper version 2.0. Peak heights from each patient were then exported to an Excel spreadsheet, which was designed to assess the ratios of each test peak relative to all other peaks for that individual. Ratios of test peaks to control peaks and control peaks to other control peaks in each patient sample were compared to the same ratios obtained for two normal individuals, which were included in each run. For normal sequences, a dosage quotient of 1.0 is expected; if a deletion or duplication is present, the dosage quotient should be 0.5 and 1.5, respectively. In a series of control experiments, we found that normal sequences gave a mean dosage quotient of 1.04 (range 0.79–1.27, standard deviation=0.06, *n*=143), deleted sequences gave a mean dosage quotient of 0.50 (range 0.34–0.67, standard deviation=0.07, *n*=110) and duplicated sequences gave a mean dosage quotient of 1.60 (range 1.32–1.73, standard deviation=0.06, *n*=60). Results were deemed acceptable if the dosage quotient for each control peak fell within the range 0.8–1.2. A deletion was scored if the mean dosage quotient of the test to internal control peaks was less than 0.7, and a duplication was scored if the mean dosage quotient was 1.3 or greater. Intermediate results were discounted and samples were repeated if the scores did not fall into the above three categories. In all cases (approximately 5% of samples), we were able to obtain a satisfactory result on repeat analysis sometimes by reducing or increasing the amount of input DNA. Deletions of multiple contiguous exons are extremely unlikely to have arisen as false positives by chance in the MLPA assay (Taylor *et al*, 2003), and thus we did not confirm these independently. Apparent deletions of a single exon, however, do need confirmation (Taylor *et al*, 2003) and so we first checked the sequence of the exon in question to see if a point mutation or microdeletion/microinsertion was present that might interfere with MLPA probe hybridisation. If the sequence was normal, the deletion was confirmed by MAPH or QF-PCR. In this series, we did not observe any false-positive results, that is, all single-exon abnormalities detected by MLPA turned out to be real copy number changes or due to exonic mutations. A typical MLPA result is shown in Figure 1

.

### dHPLC and PTT analysis

PCR products were run on a WAV-3500 Transgenomic WAVE™ machine with Navigator v1.5. 2 software and WAVE Optimized™ Buffers (Transgenomic Inc., Crewe, UK). PCR product (8 *μ*l) was injected at column temperatures selected using the Navigator melting algorithm software. In general, PCR primers (sequences available on request) were chosen to include at least 40–50 bp of flanking intron sequence for each exon. Protein truncation test analysis was performed using the standard method and primer sequences published by Plummer *et al* (1995).

### Sequencing analysis

Relevant PCR products were sequenced in both forward and reverse directions using BigDye® Terminator v1.1. or v3.1 chemistry (Applied Biosystems). Samples underwent 25 cycles of amplification (30 s at 96°C, 15 s at 50°C and 2 min at 60°C). Excess terminators were removed using DyeEx™ 2.0 spin columns (Qiagen) prior to running on an ABI 3100 genetic analyser.

### Costings

Reagent cost calculations (rounded to the nearest pound) were calculated per sample and are based on standard operational procedures employed at the Wessex Regional Genetics Laboratory, average referral rates per annum, average detection rates per gene and reagent list prices as at December 2003. Reagent costs for *hMLH1* and *hMSH2* (full mutation screen and sequence confirmation)=£56; *BRCA1/2* (PTT plus full mutation screen and sequence confirmation) £116; *APC* (full mutation screen and sequence confirmation)=£62; and MLPA=£7.

To calculate staff costs, we have used an approximate wage estimate of £10 h^−1^ (equivalent to an annual wage of £19 500) and an average hands-on time of 2 h for MLPA, 13 h for PTT plus the limited BRCA1/2 screen, 52.5 h for dHPLC analysis plus sequencing for HNPCC, an estimated 39 h for dHPLC analysis plus sequencing for a full BRCA1/2 screen and 30 h for dHPLC analysis plus sequencing for FAP. Reporting time calculations were based on a mean reporting time of 5 days for MLPA, 50 days for PTT and 90 days for dHPLC plus sequencing.

## RESULTS

### Hereditary nonpolyposis colorectal cancer

Using MLPA, we found a deletion of one or more exons of the *hMSH2* gene in seven of the 122 HNPCC patients in whom no causative mutation had been detected previously (see Table 1

**Table 1:** Mutations detected by MLPA in the Wessex cancer cohorts

**Gene**	**Mutation detected**
*hMSH2* #1	Deleted exon 1
*hMSH2* #2	Deleted exons 1–2
*hMSH2* #3	Deleted exons 1–8
*hMSH2* #4	Deleted exons 4–16
*hMSH2* #5	Deleted exons 9–16
*hMSH2* #6	Deleted exons 9–16
*hMSH2* #7	Whole gene deletion
	
*BRCA1* #1	Deleted exon 20
*BRCA1* #2	Deleted exons 9–12
*BRCA1* #3	Deleted exons 21–24
*BRCA1* #4	Deleted exons 1–17
*BRCA1* #5	Deleted exons 8–13
*BRCA1* #6	Duplicated exons 3–5
	
*BRCA2* #1	Deleted exons 1–2
	
*APC* #1	Deleted exons 4–15
*APC* #2	Deleted exons 7–15
*APC* #3	Whole gene deletion
*APC* #4	Whole gene deletion
*APC* #5	Whole gene deletion
*APC* #6	Whole gene deletion

). This equates to 4.3% of all HNPCC probands or 5.7% of probands without a known causative mutation. Four of these mutations were found in Amsterdam-positive patients (8% of all Amsterdam-positive patients; 3.3% of previously mutation-negative probands) and three were Amsterdam-negative patients (2.7% of all Amsterdam-negative patients; 2.5% of previously mutation-negative probands). One patient had a deletion of just exon 1 of the *hMSH2* gene and this was confirmed by a separate laboratory using MAPH (data not shown). No copy number changes were detected in the *hMLH1* gene.

### Breast/ovarian cancer

Of the 136 patients analysed, six copy number variants were found within the *BRCA1* gene and one copy number variant was found for *BRCA2* (5.1%. of cases that were tested and were negative in the partial mutation screen). The mutations are listed in Table 1. The individual (BRCA1 #1) with a deletion of *BRCA1* exon 20 only was confirmed in another laboratory using QF-PCR (data not shown); all the other deletions or duplications involved contiguous exons. In addition to these cases, MLPA detected apparent single-exon deletions in two breast cancer patients who were subsequently shown to have microdeletions within the MLPA probe recognition sequences: 364delT in *BRCA1* exon 6 and 983delACAG in *BRCA2* exon 9. These microdeletions prevented the efficient ligation of the MLPA probes, resulting in an apparent deletion of the whole exon. Since these mutations would have been picked up by the full mutation screens that are currently being implemented by NHS diagnostic laboratories, we did not score them as additional MLPA-detectable pathogenetic lesions in our analysis.

### FAP patients

Multiplex ligation-dependent probe amplification analysis of the 24 previously mutation-negative patients detected a gross deletion in six individuals (see Table 1), equating to 8% of all patients or 25% of patients in whom no mutation had previously been found.

### Costings

The calculations of reagent costs and staff wages are shown in Table 2

**Table 2:** Reagent costs, staff wages (in pounds sterling; overheads and equipment costs not included) and average reporting times per sample depending on whether MLPA is carried out as a prescreen or a postscreen

**Category**	**Testing order**	**Reagent cost (£)^a^**	**Staff time (£)**	**Total (£)**	**Average reporting time (days)**
All HNPCC	MLPA prescreen	61	523	584	91^b^
All HNPCC	MLPA postscreen	61	540	601	94
					
Amsterdam-positive HNPCC	MLPA prescreen	58	501	559	88^b^
Amsterdam-positive HNPCC	MLPA postscreen	59	535	594	93
					
Breast cancer (full screen)	MLPA prescreen	107	470	577	123^b^
Breast cancer (full screen)	MLPA postscreen	113	486	599	129
					
FAP	MLPA prescreen	65	292	357	89^b^
FAP	MLPA postscreen	64	299	363	92

MLPA=multiplex ligation-dependent probe amplification; HNPCC=hereditary nonpolyposis colorectal cancer; FAP=familial adenomatous polyposis coli.

a Including cost of MLPA @ £7/sample.

b Reporting time within 7 days for those cases with an MLPA-detectable copy number change.

for the different scenarios depending upon sample category and the initial mutation detection method of choice. Overheads, equipment costs and depreciation are not included. All calculations are based on our current annual referral rate for each syndrome (HNPCC, *n*=24; breast cancer, *n*=94; FAP, *n*=7). The final estimated costs are not simply the sum of the component tests due to the sequential nature of the analysis, for example, if MLPA is performed as a prescreen then those individuals who are positive will not be subject to the full mutation screen. Similarly, BRCA patients for whom mutations were identified by PTT would not be subject to further analysis, except for sequence confirmation.

For HNPCC, prescreening with MLPA results in a saving of £17 per sample for all referrals (£35 for Amsterdam-positive patients). This equates to a total financial saving per year of £408, if MLPA is the first mutation detection method used. For breast cancer patients, MLPA prescreening results in a saving of £22 per patient, a total of £2016 per year. For FAP, a saving of £6 per person can be made, a total of £49 per year.

### Reporting times

A comparison of the average reporting times (see Table 2) shows that a saving of 3 days can be obtained by prescreening all HNPCC referrals with MLPA, increasing to 5 days if testing Amsterdam-positive patients only. Although 3–6 days appears to be a minimal saving overall, each patient with an MLPA-detectable abnormality would be reported within 7 days, 81–116 days earlier than the average reporting time (Table 2). For breast cancer patients, an average of 6 days can be saved by prescreening with MLPA, and for FAP patients this figure is 3 days.

## DISCUSSION

Multiplex ligation-dependent probe amplification has rapidly gained acceptance in genetic diagnostic laboratories due to its simplicity, relatively low cost, capacity for reasonably high throughput and robustness (Di Fiore *et al*, 2004). Using MLPA, we have identified copy number variants at a frequency of 5–8% in cancer predisposition genes from families in the Wessex region. Although this is a relatively small proportion of all cases, it does represent a significant increase in the number of families for whom a causative mutation can be identified, and thus an increased number of individuals who will benefit from appropriate counselling and management. In our series, standard point mutation analysis enabled causative mutations to be identified in 25% of HNPCC families, 32% of BRCA families (partial mutation screen only) with a Manchester score of ⩾10 and 68% of FAP families. With the incorporation of MLPA in addition to point mutation screening, causative mutations were identified in 29% of HNPCC families, 37% of high-risk breast cancer families and 76% of FAP families.

Considering the nature of the copy number variants, we identified some features that deserve comment. First, for HNPCC we found seven copy number variants for *hMSH2* but none for *hMLH1*. This is in contrast to another study from the UK in which copy number variants were seen in both genes, although the overall frequency of variants was similar in both studies (Taylor *et al*, 2003). Second, copy number variants were more common for *BRCA1* (*n*=6) compared to *BRCA2* (*n*=1). Finally, four of six *APC* variants in apparently unrelated individuals involved the loss of the whole gene. Although we have not shown that these abnormalities are the same at the molecular level, they may be indicative of a local founder effect.

As expected, the proportion of HNPCC copy number variants was higher in individuals who were Amsterdam positive (8%) compared to those who were Amsterdam negative (2.7%). One unexpected benefit from this study was the detection of two small mutations at the probe binding sites (1 and 4 bp deletions) by MLPA. Although we did not score these changes as new MLPA-detectable pathogenetic lesions in our analysis, these results demonstrate that MLPA may be used to detect small mutations in addition to gross copy number changes.

The cost calculations presented here show that a modest total financial saving of around £2500 per year may be obtained by choosing MLPA as the initial mutation screening method for all hereditary cancer referrals (for a region with a population of 3 million people), as well as the additional advantage of quicker reporting times for positive individuals. The number of individuals who would benefit from the shorter reporting time equates to approximately 150–200 cases per annum in the UK.

Overall our data highlight the fact that dosage analysis is an essential tool in cancer diagnostics that should be undertaken by all laboratories, that screen for mutations in cancer predisposition genes. We have also shown that it is more appropriate to perform MLPA analysis prior to, rather than after, point mutation analysis and that reporting times can be substantially reduced for a proportion of affected individuals.
